# Supplementary material for: Researchers working from home: Benefits and challenges
Source: PLoS One. 2021 Mar 25;16(3):e0249127. doi: 10.1371/journal.pone.0249127 (PMC7993618; doi:10.1371/journal.pone.0249127)
Supplement: S1 File — (DOCX) [file pone.0249127.s001.docx]

Researchers working from home:

Benefits and challenges

Balazs Aczel^1^, Marton Kovacs^1,2^,

Barnabas Szaszi^1^, Tanja van der Lippe^3^

^1^Institute of Psychology, ELTE Eotvos Lorand University, Budapest, Hungary

^2^Doctoral School of Psychology, ELTE Eotvos Lorand University, Budapest, Hungary

^3^Department of Sociology, Utrecht University, Padualaan 14, 3584 CH, Utrecht, The Netherlands

#

**Table of Contents**

[Deviations from the preregistration](#_rvm0arais65i) 3

[Sampling](#_ag2vdx8cscr) 3

[Pilot Study](#_fvn0v4jxcq1d) 4

[Main Study](#_pr327hgq70g4) 4

[Data preprocessing](#_tt9cifelz0fz) 4

[Number of respondents in each subgroup](#_tiggist8lb9k) 4

[Efficiency ratings by subgroups](#_gn6i8hg465ro) 10

#

# Deviations from the preregistration

Our study procedure and analysis plan were preregistered at <https://osf.io/jg5bz>.

For the investigation of whether it would be possible to work from home in the future (‘Feasibility of working more from home’ section in the main text), we preregistered that we would include only those respondents who indicate that working more from home is more efficient. However, we had to realize that this exclusion rule should not be applied here as for this question the respondents had to combine the efficiency and well-being aspects of working from home and it is plausible to think that some of them might prefer to work more from home even if it came with some decrease of efficiency but an increase in well-being. Therefore, we did not apply this exclusion rule to this question.

For the additional analysis of the research efficiency for the subgroups, we preregistered that the interested subgroups will be made by the level of seniority and the data collection method. To constrain the focus of the main text, we list these results only among all the subgroups in this suppplement.

# Sampling

We distributed our online survey link among researchers in professional newsletters, university mailing lists, on social media, and by sending group-emails to authors.

We send newsletter to the members of the Society of Judgment and Decision Making and the Psychological Science Accelerator. The survey was sent out to our local faculties. We distributed the link on Twitter. As we asked recipients of the advertisement to distribute the survey among researchers, we cannot list all the forums it reached.

For the emailing, we used an email collection that we created for an earlier project. There, we collected email addresses of corresponding authors partly from scientific articles published between 2010 and 2018 in journals in the ScienceDirect database. We extracted 59,671 email addresses with a text-mining method through the Elsevier Developer API. The contact email database was created for an unfinished research project. For this project, we excluded the email addresses of authors whom we previously sent invitations for participation in other studies. Therefore, we contacted 23,849 from the database for the present study. In addition, we sent the survey to 432 email addresses of corresponding authors of scientific articles that we collected for a separate meta-research study.

# Pilot Study

We sampled 2,994 email addresses in two batches (500 and 2,494) from the contact database for the pilot study. The pilot study was sent through the Qualtrics mail sender 2020-03-15 and 2020-03-20 to the sampled email addresses.

In the pilot study, we asked the respondents how much of their work time do they spend at home during and before the pandemic in percentages. Then we asked them to list the areas of work that benefit and the areas of work that suffer when working from home. Finally, we asked the respondents whether they have home-schooling duties and their research discipline.

We received 55 responses (1.84 response rate). Two of the authors, B.A. and M.K., read through the free-text responses of benefits and downsides of working from home and used them to create the 15 aspects of research work used in the main questionnaire.

# Main Study

## Data preprocessing

During the data preprocessing, we read the free text responses of the respondents. In some cases, the content of their free text responses contradicted their choices in the survey. Therefore, we excluded one response to the question about the academic position, all the ratings of the different aspects when comparing working from home to the office from one respondent, the efficiency rating of one respondent when indicating their efficiency after the lockdown. We also changed one response from no to yes of the question that inquired whether it is possible to collect data remotely for the respondent.

## Number of respondents in each subgroup

Based on their responses to the background information questions (see the materials at <https://osf.io/8ze2g/>) we could assign each respondent to different subgroups. One respondent is a member of multiple subgroups as the respondents answered multiple background information questions. In the following table, we present the number of respondents in each subgroup for each background information question. The subgroups are listed in a decreasing order by the proportion of the subgroup. As we analyzed only complete surveys, each background question was answered by all the 704 members of our sample.

*Supplementary Table 1. Number and Proportion of Respondents in Each Subgroup*

| **Background information question** | **Subgroups** | **Number of responses** | **Proportion of the subgroup** |
| --- | --- | --- | --- |
| Academic position | full professor | 209 | 29.69 |
| Academic position | associate professor | 172 | 24.43 |
| Academic position | assistant professor | 126 | 17.90 |
| Academic position | PhD student | 72 | 10.23 |
| Academic position | postdoc | 72 | 10.23 |
| Academic position | non-academic researcher | 38 | 5.40 |
| Academic position | research assistant | 14 | 1.99 |
| Academic position | not applicable | 1 | 0.14 |
| Area of research | Social Sciences | 531 | 75.43 |
| Area of research | Life Sciences | 98 | 13.92 |
| Area of research | Other | 63 | 8.95 |
| Area of research | Formal Sciences | 5 | 0.71 |
| Area of research | Physical Sciences | 5 | 0.71 |
| Area of research | Earth & Space Sciences | 2 | 0.28 |
| Workplace type | Research and educational institute | 612 | 86.93 |
| Workplace type | Purely research institute | 57 | 8.10 |
| Workplace type | None of them | 21 | 2.98 |
| Workplace type | Purely educational institute | 14 | 1.99 |
| Data collection remotely | Yes (including if someone else does it for me) | 513 | 72.87 |
| Data collection remotely | No | 170 | 24.15 |
| Data collection remotely | not applicable | 21 | 2.98 |
| Gender | Female | 356 | 50.57 |
| Gender | Male | 338 | 48.01 |
| Gender | Prefer not to say | 9 | 1.28 |
| Gender | Other | 1 | 0.14 |
| Age | 35 - 44 | 231 | 32.81 |
| Age | 45 - 54 | 169 | 24.01 |
| Age | 25 - 34 | 150 | 21.31 |
| Age | 55 - 64 | 93 | 13.21 |
| Age | 65 - 74 | 48 | 6.82 |
| Age | Under 24 | 7 | 0.99 |
| Age | 75 - 84 | 6 | 0.85 |
| Educational duties | Yes | 566 | 80.40 |
| Educational duties | No | 138 | 19.60 |
| Team work | Yes | 373 | 52.98 |
| Team work | No | 331 | 47.02 |
| Partner working | Yes | 446 | 63.35 |
| Partner working | not applicable | 137 | 19.46 |
| Partner working | No | 121 | 17.19 |
| Living situation | Living with partner and non-adult child(ren) | 281 | 39.91 |
| Living situation | Living only with partner | 242 | 34.38 |
| Living situation | Living alone | 92 | 13.07 |
| Living situation | Other | 33 | 4.69 |
| Living situation | Living with parents or other adult family members | 25 | 3.55 |
| Living situation | Single-parent with non-adult child(ren) | 23 | 3.27 |
| Living situation | Living with non-family others | 8 | 1.14 |
| Homeschooling | No | 465 | 66.05 |
| Homeschooling | Yes | 239 | 33.95 |
| Help with childcare | not applicable | 353 | 50.14 |
| Help with childcare | No | 227 | 32.24 |
| Help with childcare | Yes | 124 | 17.61 |
| Work-home distance | <30 min | 419 | 59.52 |
| Work-home distance | 30-60 min | 196 | 27.84 |
| Work-home distance | >60 min | 89 | 12.64 |
| Home office setup | Fully equipped | 444 | 63.07 |
| Home office setup | Underequipped | 260 | 36.93 |
| Number of children | 0 | 358 | 50.85 |
| Number of children | 2 | 163 | 23.15 |
| Number of children | 1 | 128 | 18.18 |
| Number of children | 3 | 41 | 5.82 |
| Number of children | 4 | 7 | 0.99 |
| Number of children | 5 | 3 | 0.43 |
| Number of children | 12 | 1 | 0.14 |
| Number of children | 22 | 1 | 0.14 |
| Number of children | 7 | 1 | 0.14 |
| Number of children | 8 | 1 | 0.14 |

## Efficiency ratings by subgroups

The following table lists for each subgroup the numbers and proportions of respondents for each efficiency rating (less efficient, similarly efficient, more efficient) comparing their research work from home during the lockdown to before the lockdown. Only those respondents are presented in the table who indicated that they worked more from home during the lockdown than before and whose efficiency rating was not missing. The subgroups are listed in a decreasing order by the number of respondents in the subgroup.

*Supplementary Table 2. Efficiency Ratings by Each Subgroup Comparing Efficiency Before and During the Lockdown*

| **Background information question** | **Subgroups** | **Efficiency ratings** | **Number of responses to the efficiency ratings** | **Number of respondents in the subgroup** | **Proportion of the subgroup** |
| --- | --- | --- | --- | --- | --- |
| Academic position | postdoc | less efficient | 31 | 72 | 43.06 |
| Academic position | postdoc | similarly efficient | 28 | 72 | 38.89 |
| Academic position | postdoc | more efficient | 13 | 72 | 18.06 |
| Academic position | associate professor | less efficient | 87 | 165 | 52.73 |
| Academic position | associate professor | similarly efficient | 34 | 165 | 20.61 |
| Academic position | associate professor | more efficient | 44 | 165 | 26.67 |
| Academic position | assistant professor | less efficient | 71 | 117 | 60.68 |
| Academic position | assistant professor | similarly efficient | 23 | 117 | 19.66 |
| Academic position | assistant professor | more efficient | 23 | 117 | 19.66 |
| Academic position | full professor | less efficient | 79 | 195 | 40.51 |
| Academic position | full professor | similarly efficient | 79 | 195 | 40.51 |
| Academic position | full professor | more efficient | 37 | 195 | 18.97 |
| Academic position | non-academic researcher | less efficient | 11 | 33 | 33.33 |
| Academic position | non-academic researcher | similarly efficient | 8 | 33 | 24.24 |
| Academic position | non-academic researcher | more efficient | 14 | 33 | 42.42 |
| Academic position | not applicable | similarly efficient | 1 | 1 | 100.00 |
| Academic position | PhD student | less efficient | 25 | 67 | 37.31 |
| Academic position | PhD student | similarly efficient | 25 | 67 | 37.31 |
| Academic position | PhD student | more efficient | 17 | 67 | 25.37 |
| Academic position | research assistant | less efficient | 4 | 12 | 33.33 |
| Academic position | research assistant | similarly efficient | 1 | 12 | 8.33 |
| Academic position | research assistant | more efficient | 7 | 12 | 58.33 |
| Area of research | Social Sciences | less efficient | 231 | 502 | 46.02 |
| Area of research | Social Sciences | similarly efficient | 147 | 502 | 29.28 |
| Area of research | Social Sciences | more efficient | 124 | 502 | 24.70 |
| Area of research | Earth & Space Sciences | more efficient | 2 | 2 | 100.00 |
| Area of research | Life Sciences | less efficient | 45 | 89 | 50.56 |
| Area of research | Life Sciences | similarly efficient | 28 | 89 | 31.46 |
| Area of research | Life Sciences | more efficient | 16 | 89 | 17.98 |
| Area of research | Other | less efficient | 28 | 59 | 47.46 |
| Area of research | Other | similarly efficient | 21 | 59 | 35.59 |
| Area of research | Other | more efficient | 10 | 59 | 16.95 |
| Area of research | Physical Sciences | less efficient | 3 | 5 | 60.00 |
| Area of research | Physical Sciences | similarly efficient | 2 | 5 | 40.00 |
| Area of research | Formal Sciences | less efficient | 1 | 5 | 20.00 |
| Area of research | Formal Sciences | similarly efficient | 1 | 5 | 20.00 |
| Area of research | Formal Sciences | more efficient | 3 | 5 | 60.00 |
| Workplace type | Research and educational institute | less efficient | 268 | 574 | 46.69 |
| Workplace type | Research and educational institute | similarly efficient | 178 | 574 | 31.01 |
| Workplace type | Research and educational institute | more efficient | 128 | 574 | 22.30 |
| Workplace type | Purely research institute | less efficient | 27 | 55 | 49.09 |
| Workplace type | Purely research institute | similarly efficient | 13 | 55 | 23.64 |
| Workplace type | Purely research institute | more efficient | 15 | 55 | 27.27 |
| Workplace type | None of them | less efficient | 4 | 19 | 21.05 |
| Workplace type | None of them | similarly efficient | 5 | 19 | 26.32 |
| Workplace type | None of them | more efficient | 10 | 19 | 52.63 |
| Workplace type | Purely educational institute | less efficient | 9 | 14 | 64.29 |
| Workplace type | Purely educational institute | similarly efficient | 3 | 14 | 21.43 |
| Workplace type | Purely educational institute | more efficient | 2 | 14 | 14.29 |
| Data collection remotely | No | less efficient | 86 | 158 | 54.43 |
| Data collection remotely | No | similarly efficient | 46 | 158 | 29.11 |
| Data collection remotely | No | more efficient | 26 | 158 | 16.46 |
| Data collection remotely | Yes (including if someone else does it for me) | less efficient | 218 | 485 | 44.95 |
| Data collection remotely | Yes (including if someone else does it for me) | similarly efficient | 144 | 485 | 29.69 |
| Data collection remotely | Yes (including if someone else does it for me) | more efficient | 123 | 485 | 25.36 |
| Data collection remotely | not applicable | less efficient | 4 | 19 | 21.05 |
| Data collection remotely | not applicable | similarly efficient | 9 | 19 | 47.37 |
| Data collection remotely | not applicable | more efficient | 6 | 19 | 31.58 |
| Gender | Male | less efficient | 153 | 317 | 48.26 |
| Gender | Male | similarly efficient | 97 | 317 | 30.60 |
| Gender | Male | more efficient | 67 | 317 | 21.14 |
| Gender | Female | less efficient | 150 | 337 | 44.51 |
| Gender | Female | similarly efficient | 101 | 337 | 29.97 |
| Gender | Female | more efficient | 86 | 337 | 25.52 |
| Gender | Prefer not to say | less efficient | 5 | 7 | 71.43 |
| Gender | Prefer not to say | similarly efficient | 1 | 7 | 14.29 |
| Gender | Prefer not to say | more efficient | 1 | 7 | 14.29 |
| Gender | Other | more efficient | 1 | 1 | 100.00 |
| Age | 25 - 34 | less efficient | 58 | 144 | 40.28 |
| Age | 25 - 34 | similarly efficient | 44 | 144 | 30.56 |
| Age | 25 - 34 | more efficient | 42 | 144 | 29.17 |
| Age | Under 24 | less efficient | 2 | 7 | 28.57 |
| Age | Under 24 | similarly efficient | 3 | 7 | 42.86 |
| Age | Under 24 | more efficient | 2 | 7 | 28.57 |
| Age | 35 - 44 | less efficient | 118 | 216 | 54.63 |
| Age | 35 - 44 | similarly efficient | 56 | 216 | 25.93 |
| Age | 35 - 44 | more efficient | 42 | 216 | 19.44 |
| Age | 55 - 64 | less efficient | 42 | 90 | 46.67 |
| Age | 55 - 64 | similarly efficient | 31 | 90 | 34.44 |
| Age | 55 - 64 | more efficient | 17 | 90 | 18.89 |
| Age | 45 - 54 | less efficient | 77 | 162 | 47.53 |
| Age | 45 - 54 | similarly efficient | 45 | 162 | 27.78 |
| Age | 45 - 54 | more efficient | 40 | 162 | 24.69 |
| Age | 65 - 74 | less efficient | 10 | 38 | 26.32 |
| Age | 65 - 74 | similarly efficient | 19 | 38 | 50.00 |
| Age | 65 - 74 | more efficient | 9 | 38 | 23.68 |
| Age | 75 - 84 | less efficient | 1 | 5 | 20.00 |
| Age | 75 - 84 | similarly efficient | 1 | 5 | 20.00 |
| Age | 75 - 84 | more efficient | 3 | 5 | 60.00 |
| Educational duties | No | less efficient | 44 | 121 | 36.36 |
| Educational duties | No | similarly efficient | 40 | 121 | 33.06 |
| Educational duties | No | more efficient | 37 | 121 | 30.58 |
| Educational duties | Yes | less efficient | 264 | 541 | 48.80 |
| Educational duties | Yes | similarly efficient | 159 | 541 | 29.39 |
| Educational duties | Yes | more efficient | 118 | 541 | 21.81 |
| Team work | Yes | less efficient | 157 | 355 | 44.23 |
| Team work | Yes | similarly efficient | 111 | 355 | 31.27 |
| Team work | Yes | more efficient | 87 | 355 | 24.51 |
| Team work | No | less efficient | 151 | 307 | 49.19 |
| Team work | No | similarly efficient | 88 | 307 | 28.66 |
| Team work | No | more efficient | 68 | 307 | 22.15 |
| Partner working | Yes | less efficient | 188 | 418 | 44.98 |
| Partner working | Yes | similarly efficient | 128 | 418 | 30.62 |
| Partner working | Yes | more efficient | 102 | 418 | 24.40 |
| Partner working | No | less efficient | 56 | 114 | 49.12 |
| Partner working | No | similarly efficient | 31 | 114 | 27.19 |
| Partner working | No | more efficient | 27 | 114 | 23.68 |
| Partner working | not applicable | less efficient | 64 | 130 | 49.23 |
| Partner working | not applicable | similarly efficient | 40 | 130 | 30.77 |
| Partner working | not applicable | more efficient | 26 | 130 | 20.00 |
| Living situation | Living only with partner | less efficient | 82 | 223 | 36.77 |
| Living situation | Living only with partner | similarly efficient | 85 | 223 | 38.12 |
| Living situation | Living only with partner | more efficient | 56 | 223 | 25.11 |
| Living situation | Living with partner and non-adult child(ren) | less efficient | 153 | 269 | 56.88 |
| Living situation | Living with partner and non-adult child(ren) | similarly efficient | 61 | 269 | 22.68 |
| Living situation | Living with partner and non-adult child(ren) | more efficient | 55 | 269 | 20.45 |
| Living situation | Living alone | less efficient | 33 | 87 | 37.93 |
| Living situation | Living alone | similarly efficient | 28 | 87 | 32.18 |
| Living situation | Living alone | more efficient | 26 | 87 | 29.89 |
| Living situation | Other | less efficient | 12 | 30 | 40.00 |
| Living situation | Other | similarly efficient | 11 | 30 | 36.67 |
| Living situation | Other | more efficient | 7 | 30 | 23.33 |
| Living situation | Living with parents or other adult family members | less efficient | 10 | 25 | 40.00 |
| Living situation | Living with parents or other adult family members | similarly efficient | 8 | 25 | 32.00 |
| Living situation | Living with parents or other adult family members | more efficient | 7 | 25 | 28.00 |
| Living situation | Single-parent with non-adult child(ren) | less efficient | 15 | 21 | 71.43 |
| Living situation | Single-parent with non-adult child(ren) | similarly efficient | 4 | 21 | 19.05 |
| Living situation | Single-parent with non-adult child(ren) | more efficient | 2 | 21 | 9.52 |
| Living situation | Living with non-family others | less efficient | 3 | 7 | 42.86 |
| Living situation | Living with non-family others | similarly efficient | 2 | 7 | 28.57 |
| Living situation | Living with non-family others | more efficient | 2 | 7 | 28.57 |
| Homeschooling | No | less efficient | 173 | 436 | 39.68 |
| Homeschooling | No | similarly efficient | 147 | 436 | 33.72 |
| Homeschooling | No | more efficient | 116 | 436 | 26.61 |
| Homeschooling | Yes | less efficient | 135 | 226 | 59.73 |
| Homeschooling | Yes | similarly efficient | 52 | 226 | 23.01 |
| Homeschooling | Yes | more efficient | 39 | 226 | 17.26 |
| Help with childcare | not applicable | less efficient | 123 | 329 | 37.39 |
| Help with childcare | not applicable | similarly efficient | 114 | 329 | 34.65 |
| Help with childcare | not applicable | more efficient | 92 | 329 | 27.96 |
| Help with childcare | No | less efficient | 120 | 214 | 56.07 |
| Help with childcare | No | similarly efficient | 57 | 214 | 26.64 |
| Help with childcare | No | more efficient | 37 | 214 | 17.29 |
| Help with childcare | Yes | less efficient | 65 | 119 | 54.62 |
| Help with childcare | Yes | similarly efficient | 28 | 119 | 23.53 |
| Help with childcare | Yes | more efficient | 26 | 119 | 21.85 |
| Work-home distance | 30-60 min | less efficient | 75 | 187 | 40.11 |
| Work-home distance | 30-60 min | similarly efficient | 58 | 187 | 31.02 |
| Work-home distance | 30-60 min | more efficient | 54 | 187 | 28.88 |
| Work-home distance | >60 min | less efficient | 26 | 80 | 32.50 |
| Work-home distance | >60 min | similarly efficient | 29 | 80 | 36.25 |
| Work-home distance | >60 min | more efficient | 25 | 80 | 31.25 |
| Work-home distance | <30 min | less efficient | 207 | 395 | 52.41 |
| Work-home distance | <30 min | similarly efficient | 112 | 395 | 28.35 |
| Work-home distance | <30 min | more efficient | 76 | 395 | 19.24 |
| Home office setup | Fully equipped | less efficient | 175 | 417 | 41.97 |
| Home office setup | Fully equipped | similarly efficient | 130 | 417 | 31.18 |
| Home office setup | Fully equipped | more efficient | 112 | 417 | 26.86 |
| Home office setup | Underequipped | less efficient | 133 | 245 | 54.29 |
| Home office setup | Underequipped | similarly efficient | 69 | 245 | 28.16 |
| Home office setup | Underequipped | more efficient | 43 | 245 | 17.55 |
| Number of children | 0 | less efficient | 122 | 331 | 36.86 |
| Number of children | 0 | similarly efficient | 120 | 331 | 36.25 |
| Number of children | 0 | more efficient | 89 | 331 | 26.89 |
| Number of children | 7 | more efficient | 1 | 1 | 100.00 |
| Number of children | 2 | less efficient | 91 | 152 | 59.87 |
| Number of children | 2 | similarly efficient | 34 | 152 | 22.37 |
| Number of children | 2 | more efficient | 27 | 152 | 17.76 |
| Number of children | 1 | less efficient | 63 | 126 | 50.00 |
| Number of children | 1 | similarly efficient | 38 | 126 | 30.16 |
| Number of children | 1 | more efficient | 25 | 126 | 19.84 |
| Number of children | 4 | less efficient | 5 | 7 | 71.43 |
| Number of children | 4 | more efficient | 2 | 7 | 28.57 |
| Number of children | 3 | less efficient | 23 | 40 | 57.50 |
| Number of children | 3 | similarly efficient | 7 | 40 | 17.50 |
| Number of children | 3 | more efficient | 10 | 40 | 25.00 |
| Number of children | 12 | more efficient | 1 | 1 | 100.00 |
| Number of children | 22 | less efficient | 1 | 1 | 100.00 |
| Number of children | 8 | less efficient | 1 | 1 | 100.00 |
| Number of children | 5 | less efficient | 2 | 2 | 100.00 |

The following table lists for each subgroup the numbers and proportions of respondents for each efficiency rating (less efficient, similarly efficient, more efficient) indicating their expected efficiency for working from home after the lockdown. Only those respondents are presented in the table who indicated that they did not work full-time from home prior to the pandemic. The subgroups are listed in a decreasing order by the number of respondents in the subgroup.

*Supplementary Table 3. Efficiency Ratings by Each Subgroup Comparing Efficiency During and After the Lockdown*

| **Background information question** | **Subgroups** | **Efficiency ratings** | **Number of responses to the efficiency ratings** | **Number of respondents in the subgroup** | **Proportion of the subgroup** |
| --- | --- | --- | --- | --- | --- |
| Academic position | postdoc | less efficient | 23 | 72 | 31.94 |
| Academic position | postdoc | similarly efficient | 27 | 72 | 37.50 |
| Academic position | postdoc | more efficient | 22 | 72 | 30.56 |
| Academic position | associate professor | less efficient | 52 | 168 | 30.95 |
| Academic position | associate professor | similarly efficient | 64 | 168 | 38.10 |
| Academic position | associate professor | more efficient | 52 | 168 | 30.95 |
| Academic position | assistant professor | less efficient | 40 | 124 | 32.26 |
| Academic position | assistant professor | similarly efficient | 48 | 124 | 38.71 |
| Academic position | assistant professor | more efficient | 36 | 124 | 29.03 |
| Academic position | full professor | less efficient | 60 | 202 | 29.70 |
| Academic position | full professor | similarly efficient | 97 | 202 | 48.02 |
| Academic position | full professor | more efficient | 45 | 202 | 22.28 |
| Academic position | non-academic researcher | less efficient | 5 | 34 | 14.71 |
| Academic position | non-academic researcher | similarly efficient | 12 | 34 | 35.29 |
| Academic position | non-academic researcher | more efficient | 17 | 34 | 50.00 |
| Academic position | not applicable | more efficient | 1 | 1 | 100.00 |
| Academic position | PhD student | less efficient | 18 | 69 | 26.09 |
| Academic position | PhD student | similarly efficient | 31 | 69 | 44.93 |
| Academic position | PhD student | more efficient | 20 | 69 | 28.99 |
| Academic position | research assistant | less efficient | 3 | 14 | 21.43 |
| Academic position | research assistant | similarly efficient | 3 | 14 | 21.43 |
| Academic position | research assistant | more efficient | 8 | 14 | 57.14 |
| Area of research | Social Sciences | less efficient | 149 | 514 | 28.99 |
| Area of research | Social Sciences | similarly efficient | 212 | 514 | 41.25 |
| Area of research | Social Sciences | more efficient | 153 | 514 | 29.77 |
| Area of research | Earth & Space Sciences | similarly efficient | 1 | 2 | 50.00 |
| Area of research | Earth & Space Sciences | more efficient | 1 | 2 | 50.00 |
| Area of research | Life Sciences | less efficient | 29 | 96 | 30.21 |
| Area of research | Life Sciences | similarly efficient | 41 | 96 | 42.71 |
| Area of research | Life Sciences | more efficient | 26 | 96 | 27.08 |
| Area of research | Other | less efficient | 20 | 62 | 32.26 |
| Area of research | Other | similarly efficient | 25 | 62 | 40.32 |
| Area of research | Other | more efficient | 17 | 62 | 27.42 |
| Area of research | Physical Sciences | less efficient | 2 | 5 | 40.00 |
| Area of research | Physical Sciences | similarly efficient | 2 | 5 | 40.00 |
| Area of research | Physical Sciences | more efficient | 1 | 5 | 20.00 |
| Area of research | Formal Sciences | less efficient | 1 | 5 | 20.00 |
| Area of research | Formal Sciences | similarly efficient | 1 | 5 | 20.00 |
| Area of research | Formal Sciences | more efficient | 3 | 5 | 60.00 |
| Workplace type | Research and educational institute | less efficient | 182 | 594 | 30.64 |
| Workplace type | Research and educational institute | similarly efficient | 247 | 594 | 41.58 |
| Workplace type | Research and educational institute | more efficient | 165 | 594 | 27.78 |
| Workplace type | Purely research institute | less efficient | 14 | 56 | 25.00 |
| Workplace type | Purely research institute | similarly efficient | 24 | 56 | 42.86 |
| Workplace type | Purely research institute | more efficient | 18 | 56 | 32.14 |
| Workplace type | None of them | less efficient | 2 | 20 | 10.00 |
| Workplace type | None of them | similarly efficient | 5 | 20 | 25.00 |
| Workplace type | None of them | more efficient | 13 | 20 | 65.00 |
| Workplace type | Purely educational institute | less efficient | 3 | 14 | 21.43 |
| Workplace type | Purely educational institute | similarly efficient | 6 | 14 | 42.86 |
| Workplace type | Purely educational institute | more efficient | 5 | 14 | 35.71 |
| Data collection remotely | No | less efficient | 62 | 168 | 36.90 |
| Data collection remotely | No | similarly efficient | 72 | 168 | 42.86 |
| Data collection remotely | No | more efficient | 34 | 168 | 20.24 |
| Data collection remotely | Yes (including if someone else does it for me) | less efficient | 136 | 495 | 27.47 |
| Data collection remotely | Yes (including if someone else does it for me) | similarly efficient | 200 | 495 | 40.40 |
| Data collection remotely | Yes (including if someone else does it for me) | more efficient | 159 | 495 | 32.12 |
| Data collection remotely | not applicable | less efficient | 3 | 21 | 14.29 |
| Data collection remotely | not applicable | similarly efficient | 10 | 21 | 47.62 |
| Data collection remotely | not applicable | more efficient | 8 | 21 | 38.10 |
| Gender | Male | less efficient | 106 | 331 | 32.02 |
| Gender | Male | similarly efficient | 137 | 331 | 41.39 |
| Gender | Male | more efficient | 88 | 331 | 26.59 |
| Gender | Female | less efficient | 90 | 344 | 26.16 |
| Gender | Female | similarly efficient | 142 | 344 | 41.28 |
| Gender | Female | more efficient | 112 | 344 | 32.56 |
| Gender | Prefer not to say | less efficient | 5 | 8 | 62.50 |
| Gender | Prefer not to say | similarly efficient | 3 | 8 | 37.50 |
| Gender | Other | more efficient | 1 | 1 | 100.00 |
| Age | 25 - 34 | less efficient | 43 | 149 | 28.86 |
| Age | 25 - 34 | similarly efficient | 54 | 149 | 36.24 |
| Age | 25 - 34 | more efficient | 52 | 149 | 34.90 |
| Age | Under 24 | less efficient | 1 | 7 | 14.29 |
| Age | Under 24 | similarly efficient | 3 | 7 | 42.86 |
| Age | Under 24 | more efficient | 3 | 7 | 42.86 |
| Age | 35 - 44 | less efficient | 71 | 226 | 31.42 |
| Age | 35 - 44 | similarly efficient | 99 | 226 | 43.81 |
| Age | 35 - 44 | more efficient | 56 | 226 | 24.78 |
| Age | 55 - 64 | less efficient | 25 | 90 | 27.78 |
| Age | 55 - 64 | similarly efficient | 42 | 90 | 46.67 |
| Age | 55 - 64 | more efficient | 23 | 90 | 25.56 |
| Age | 45 - 54 | less efficient | 49 | 163 | 30.06 |
| Age | 45 - 54 | similarly efficient | 60 | 163 | 36.81 |
| Age | 45 - 54 | more efficient | 54 | 163 | 33.13 |
| Age | 65 - 74 | less efficient | 10 | 44 | 22.73 |
| Age | 65 - 74 | similarly efficient | 23 | 44 | 52.27 |
| Age | 65 - 74 | more efficient | 11 | 44 | 25.00 |
| Age | 75 - 84 | less efficient | 2 | 5 | 40.00 |
| Age | 75 - 84 | similarly efficient | 1 | 5 | 20.00 |
| Age | 75 - 84 | more efficient | 2 | 5 | 40.00 |
| Educational duties | No | less efficient | 33 | 132 | 25.00 |
| Educational duties | No | similarly efficient | 51 | 132 | 38.64 |
| Educational duties | No | more efficient | 48 | 132 | 36.36 |
| Educational duties | Yes | less efficient | 168 | 552 | 30.43 |
| Educational duties | Yes | similarly efficient | 231 | 552 | 41.85 |
| Educational duties | Yes | more efficient | 153 | 552 | 27.72 |
| Team work | Yes | less efficient | 107 | 362 | 29.56 |
| Team work | Yes | similarly efficient | 151 | 362 | 41.71 |
| Team work | Yes | more efficient | 104 | 362 | 28.73 |
| Team work | No | less efficient | 94 | 322 | 29.19 |
| Team work | No | similarly efficient | 131 | 322 | 40.68 |
| Team work | No | more efficient | 97 | 322 | 30.12 |
| Partner working | Yes | less efficient | 111 | 431 | 25.75 |
| Partner working | Yes | similarly efficient | 184 | 431 | 42.69 |
| Partner working | Yes | more efficient | 136 | 431 | 31.55 |
| Partner working | No | less efficient | 38 | 116 | 32.76 |
| Partner working | No | similarly efficient | 47 | 116 | 40.52 |
| Partner working | No | more efficient | 31 | 116 | 26.72 |
| Partner working | not applicable | less efficient | 52 | 137 | 37.96 |
| Partner working | not applicable | similarly efficient | 51 | 137 | 37.23 |
| Partner working | not applicable | more efficient | 34 | 137 | 24.82 |
| Living situation | Living only with partner | less efficient | 52 | 231 | 22.51 |
| Living situation | Living only with partner | similarly efficient | 111 | 231 | 48.05 |
| Living situation | Living only with partner | more efficient | 68 | 231 | 29.44 |
| Living situation | Living with partner and non-adult child(ren) | less efficient | 82 | 272 | 30.15 |
| Living situation | Living with partner and non-adult child(ren) | similarly efficient | 105 | 272 | 38.60 |
| Living situation | Living with partner and non-adult child(ren) | more efficient | 85 | 272 | 31.25 |
| Living situation | Living alone | less efficient | 29 | 92 | 31.52 |
| Living situation | Living alone | similarly efficient | 36 | 92 | 39.13 |
| Living situation | Living alone | more efficient | 27 | 92 | 29.35 |
| Living situation | Other | less efficient | 11 | 33 | 33.33 |
| Living situation | Other | similarly efficient | 13 | 33 | 39.39 |
| Living situation | Other | more efficient | 9 | 33 | 27.27 |
| Living situation | Living with parents or other adult family members | less efficient | 10 | 25 | 40.00 |
| Living situation | Living with parents or other adult family members | similarly efficient | 7 | 25 | 28.00 |
| Living situation | Living with parents or other adult family members | more efficient | 8 | 25 | 32.00 |
| Living situation | Single-parent with non-adult child(ren) | less efficient | 13 | 23 | 56.52 |
| Living situation | Single-parent with non-adult child(ren) | similarly efficient | 8 | 23 | 34.78 |
| Living situation | Single-parent with non-adult child(ren) | more efficient | 2 | 23 | 8.70 |
| Living situation | Living with non-family others | less efficient | 4 | 8 | 50.00 |
| Living situation | Living with non-family others | similarly efficient | 2 | 8 | 25.00 |
| Living situation | Living with non-family others | more efficient | 2 | 8 | 25.00 |
| Homeschooling | No | less efficient | 128 | 454 | 28.19 |
| Homeschooling | No | similarly efficient | 187 | 454 | 41.19 |
| Homeschooling | No | more efficient | 139 | 454 | 30.62 |
| Homeschooling | Yes | less efficient | 73 | 230 | 31.74 |
| Homeschooling | Yes | similarly efficient | 95 | 230 | 41.30 |
| Homeschooling | Yes | more efficient | 62 | 230 | 26.96 |
| Help with childcare | not applicable | less efficient | 91 | 344 | 26.45 |
| Help with childcare | not applicable | similarly efficient | 151 | 344 | 43.90 |
| Help with childcare | not applicable | more efficient | 102 | 344 | 29.65 |
| Help with childcare | No | less efficient | 72 | 217 | 33.18 |
| Help with childcare | No | similarly efficient | 84 | 217 | 38.71 |
| Help with childcare | No | more efficient | 61 | 217 | 28.11 |
| Help with childcare | Yes | less efficient | 38 | 123 | 30.89 |
| Help with childcare | Yes | similarly efficient | 47 | 123 | 38.21 |
| Help with childcare | Yes | more efficient | 38 | 123 | 30.89 |
| Work-home distance | 30-60 min | less efficient | 38 | 189 | 20.11 |
| Work-home distance | 30-60 min | similarly efficient | 83 | 189 | 43.92 |
| Work-home distance | 30-60 min | more efficient | 68 | 189 | 35.98 |
| Work-home distance | >60 min | less efficient | 15 | 85 | 17.65 |
| Work-home distance | >60 min | similarly efficient | 39 | 85 | 45.88 |
| Work-home distance | >60 min | more efficient | 31 | 85 | 36.47 |
| Work-home distance | <30 min | less efficient | 148 | 410 | 36.10 |
| Work-home distance | <30 min | similarly efficient | 160 | 410 | 39.02 |
| Work-home distance | <30 min | more efficient | 102 | 410 | 24.88 |
| Home office setup | Fully equipped | less efficient | 100 | 432 | 23.15 |
| Home office setup | Fully equipped | similarly efficient | 185 | 432 | 42.82 |
| Home office setup | Fully equipped | more efficient | 147 | 432 | 34.03 |
| Home office setup | Underequipped | less efficient | 101 | 252 | 40.08 |
| Home office setup | Underequipped | similarly efficient | 97 | 252 | 38.49 |
| Home office setup | Underequipped | more efficient | 54 | 252 | 21.43 |
| Number of children | 0 | less efficient | 91 | 347 | 26.22 |
| Number of children | 0 | similarly efficient | 154 | 347 | 44.38 |
| Number of children | 0 | more efficient | 102 | 347 | 29.39 |
| Number of children | 7 | more efficient | 1 | 1 | 100.00 |
| Number of children | 3 | less efficient | 11 | 37 | 29.73 |
| Number of children | 3 | similarly efficient | 16 | 37 | 43.24 |
| Number of children | 3 | more efficient | 10 | 37 | 27.03 |
| Number of children | 1 | less efficient | 43 | 127 | 33.86 |
| Number of children | 1 | similarly efficient | 49 | 127 | 38.58 |
| Number of children | 1 | more efficient | 35 | 127 | 27.56 |
| Number of children | 2 | less efficient | 53 | 160 | 33.12 |
| Number of children | 2 | similarly efficient | 58 | 160 | 36.25 |
| Number of children | 2 | more efficient | 49 | 160 | 30.63 |
| Number of children | 4 | similarly efficient | 3 | 6 | 50.00 |
| Number of children | 4 | more efficient | 3 | 6 | 50.00 |
| Number of children | 12 | more efficient | 1 | 1 | 100.00 |
| Number of children | 22 | similarly efficient | 1 | 1 | 100.00 |
| Number of children | 5 | less efficient | 2 | 3 | 66.67 |
| Number of children | 5 | similarly efficient | 1 | 3 | 33.33 |
| Number of children | 8 | less efficient | 1 | 1 | 100.00 |
