# Supplementary material for: Researchers working from home: Benefits and challenges
Source: PLoS One. 2021 Mar 25;16(3):e0249127. doi: 10.1371/journal.pone.0249127 (PMC7993618; doi:10.1371/journal.pone.0249127)
Supplement: Attachment — Submitted filename: Response to Reviewers.pdf [file pone.0249127.s003.pdf]

PONE-D-20-30010

Researchers working from home: Benefits and challenges

Dear Dr. Johnson Cheung,

We are happy to submit a revised version of our manuscript to PLOS One.

We would like to thank you and the reviewer for their comments and suggestions.

Below, you can find the detailed responses to all comments in bold.

Kind regards,

Balazs Aczel, on behalf of all co-authors

Comments to the Author

Reviewer #1

Comment 1

Overall, the authors have revised the manuscript to increase clarity and improve understanding of the contributions that this research provides regarding the future outlook for academics working from home. I have a few minor comments:

Limitations: This revised document brings to light the fact that 1) we do not know how the transition to working from home differs between countries since country was not a survey question (which could differ significantly given a number of social and technological/infrastructure factors), and 2) since the analysis only included descriptive statistics there is great potential in learning more from this dataset – and it is wonderful that the dataset will be publicly available. I do recommend adding a statement on limitations, both because it is a best practice, and because it shows that the authors have been thoughtful about the limits of their current analysis.

Reply 1

We have now included a statement of limitations regarding the missing information on country of residence and made it more clear in the limitations section that the present study was only exploratory.

Comment 2

Results – Recommend providing a demographics table in the manuscript that displays sample size and % for the information described in the “background information” section. I appreciate the authors’ response to this request, but suggest that as a standard practice a shortened version of the key demographics could be provided in a table within the text, and

the remainder of the demographics table could be in the supplemental material (having these results within the table is standard in my field since it provides the background information necessary for academics to easily understand the full scope of the results). In response to the question of length, I would suggest that the paragraph that lists the % of respondents who were male/female, etc. could be shortened and simply refer to the table instead.

Reply 2

We have now included the key demographics as a table (Table 2) in the manuscript in addition to the full summary of all the responses in the Supplementary information.

Comment 3

Figure 1 – recommend revising the X axis to show sample size, and the bar labels to show % to increase clarity of results. The authors responded that this change was made in the revision, but I could not find the updated figure in the revised document.

Reply 3

We made sure that all the figures are updated and uploaded with this submission.
